# Supplementary material for: Performance of the Access Bio/CareStart rapid diagnostic test for the detection of glucose-6-phosphate dehydrogenase deficiency: A systematic review and meta-analysis
Source: PLoS Med. 2019 Dec 13;16(12):e1002992. doi: 10.1371/journal.pmed.1002992 (PMC6910667; doi:10.1371/journal.pmed.1002992)
Supplement: S1 Table — (DOCX) [file pmed.1002992.s002.docx]

**S1 Table: Test methods applied**

|  | **Spectrophotometry** | | | | **CSG** | | |  |
| --- | --- | --- | --- | --- | --- | --- | --- | --- |
| **Article** | Spectrophotometer | Temp. controlled (Y/N) | Duplicate testing (Y/N) | Daily controls? (Y/N) | Invalid result recorded? (Y/N) | CSG done by two readers? (Y/N) | CSG reader blinded for spec.? (Y/N) | Max. time between CSG and spec. (hours) |
| Bancone, 2015 [21] | Shimadzu UV 1800 (Shimaduz, Japan) | Y | Y | Y | Y | Y | Y | 24 |
| Espino, 2016 [29] | Mindray BA-88A (Mindray, China) | Y | N | Y | N | Y | Y | Capillary: 48  Venous: 144 |
| Henriques, 2018 [20] | Shimadzu UV 1800 (Shimadzu, Japan) | Y | Y | Y | Y | Y | Y | 48 |
| Oo, 2016 [30] | Shimadzu UV mini-1240 (Shimadzu, Japan) | Y | Y | Y | Y | Y | Y | 6 |
| Roca-Feltrer, 2014 [31] | Integra 400 analyzer (Roche Diagnostic, France) | Y | N | Y | Y | Y | Y | 24 |
| Roh, 2016, Uganda [33] | Humastar 80 (Human diagnostics, Germany) | Y | N | Y | Y | N | Y | 24 |
| Satyagraha, 2016 [32] | Shimadzu UV 1800 (Shimadzu, Japan) | Y | Y* | Y | Y | Y | Y | 72 |
| von Fricken, 2014 [34] | Thermo Scientific GENESYS 20 (Thermo Scientific, USA) | N | N | Y | N | Y | Y | 48 |

Y=yes, N=no, DK=don’t know, *Triplicate testing
