# Supplementary material for: Performance of the Access Bio/CareStart rapid diagnostic test for the detection of glucose-6-phosphate dehydrogenase deficiency: A systematic review and meta-analysis
Source: PLoS Med. 2019 Dec 13;16(12):e1002992. doi: 10.1371/journal.pmed.1002992 (PMC6910667; doi:10.1371/journal.pmed.1002992)
Supplement: S2 Table — (DOCX) [file pmed.1002992.s003.docx]

**S2 Table:** Details on studies not included

| **Article** | **Blood** | **Country** | **Sample size and study population** | **n with malaria (%)** | **Females (%)** | **Males (%)** | **100% G6PD activity in U/gHb; definition of G6PDd in U/gHb (%)** | **n with G6PDd (%)** | **Sensitivity of CSG in % (95CI)*** | **Specificity of CSG in % (95CI)*** |
| --- | --- | --- | --- | --- | --- | --- | --- | --- | --- | --- |
| Brito, 2015 [35] | Venous | Brazil | 674 Males attending health care facility | 320 (47.5) | 0 (0.0) | 674 (100.0) | 6.6 U/gHb, 2.0 U/gHb (30%) | 13 / 674 (1.9) | Malaria patients:  50.0 (21.5 – 78.5), Non-malaria patients: 80.0 (37.3-96.4) | Malaria patients:  98.4 (96.3 – 99.3), Non-malaria patients: 98.3 (96.3-99.2) |
| Abdul-Ghani, 2016 [36] | Venous | Yemen | 400 children from a x-sectional survey | 0 (0) | 181 (45.3) | 219 (54.8) | 5.0 U/gHb; 1.5 U/gHb (30%) | 37 / 400 (9.3) | 97.4 (87.0 – 100.0) | 96.4 (94.0 – 98.0) |
| Adu-Gyasi, 2015 [37] | Capillary | Ghana | 206 purposively selected participants | 54 (26.2) | 87 (42.2) | 119 (57.8) | 5.5 U/gHb; 4.1 U/gHb (74.5%) | 67 / 206 (32.5) | 100.0 (94.7 – 100.0) | 72.1 (64.1 – 79.2) |

*Derived from article, not re-calculated
