# Supplementary material for: Performance of the Access Bio/CareStart rapid diagnostic test for the detection of glucose-6-phosphate dehydrogenase deficiency: A systematic review and meta-analysis
Source: PLoS Med. 2019 Dec 13;16(12):e1002992. doi: 10.1371/journal.pmed.1002992 (PMC6910667; doi:10.1371/journal.pmed.1002992)
Supplement: S3 Table — (DOCX) [file pmed.1002992.s004.docx]

**S3 Table:** Contact details from which data were obtained

| **Title** | **Contact name** | **Email** |
| --- | --- | --- |
| Screening for Glucose-6-Phosphate Dehydrogenase Deficiency Using Three Detection Methods: A Cross-Sectional Survey in Southwestern Uganda[44] | Martina Wade, Yale School of Public Health, USA | Martina.wade@yale.edu |
| Assessment of Point-of-Care Diagnostics for G6PD Deficiency in Malaria Endemic Rural Eastern Indonesia [32] | Lydia Visita Panggalo, Eijkmans Institute for Molecular Biology, Indonesia | lydiavisita@eijkman.go.id |
| Field Trial Evaluation of the Performances of Point-of-Care Tests for Screening G6PD Deficiency in Cambodia [31] | Eric Legrand , Institute Pasteur, France | eric.legrand@pasteur.fr |
| Performance of the CareStart Glucose-6-Phosphate Dehydrogenase (G6PD) Rapid Diagnostic Test in Gressier, Haiti [34] | Bernard Okech, George Mason University, USA | bokech@ufl.edu |
| Comparison of Three Screening Test Kits for G6PD Enzyme Deficiency: Implications for Its Use in the Radical Cure of Vivax Malaria in Remote and Resource-Poor Areas in the Philippines [29] | Jobel Sornillo, Research Institute for Tropical Medicine, The Philippines | j.sornillo@gmail.com |
| Validation of G6PD Point-of-Care Tests among Healthy Volunteers in Yangon, Myanmar [30] | Rita Chanviriyavuth , Mahidol Oxford Tropical Medicine Research Unit, Thailand | rita@tropmedres.ac |
| Suitability of Capillary Blood for Quantitative Assessment of G6PD Activity and Performances of G6PD Point-of-Care Tests [21] | Rita Chanviriyavuth , Mahidol Oxford Tropical Medicine Research Unit, Thailand | rita@tropmedres.ac |
| Comparison of glucose-6 phosphate dehydrogenase status by fluorescent spot test and rapid diagnostic test in Lao PDR and Cambodia"[20] | Rita Chanviriyavuth , Mahidol Oxford Tropical Medicine Research Unit, Thailand | rita@tropmedres.ac |
