# Supplementary figures and images for: Performance of the Access Bio/CareStart rapid diagnostic test for the detection of glucose-6-phosphate dehydrogenase deficiency: A systematic review and meta-analysis
Source: PLoS Med. 2019 Dec 13;16(12):e1002992. doi: 10.1371/journal.pmed.1002992 (PMC6910667; doi:10.1371/journal.pmed.1002992)

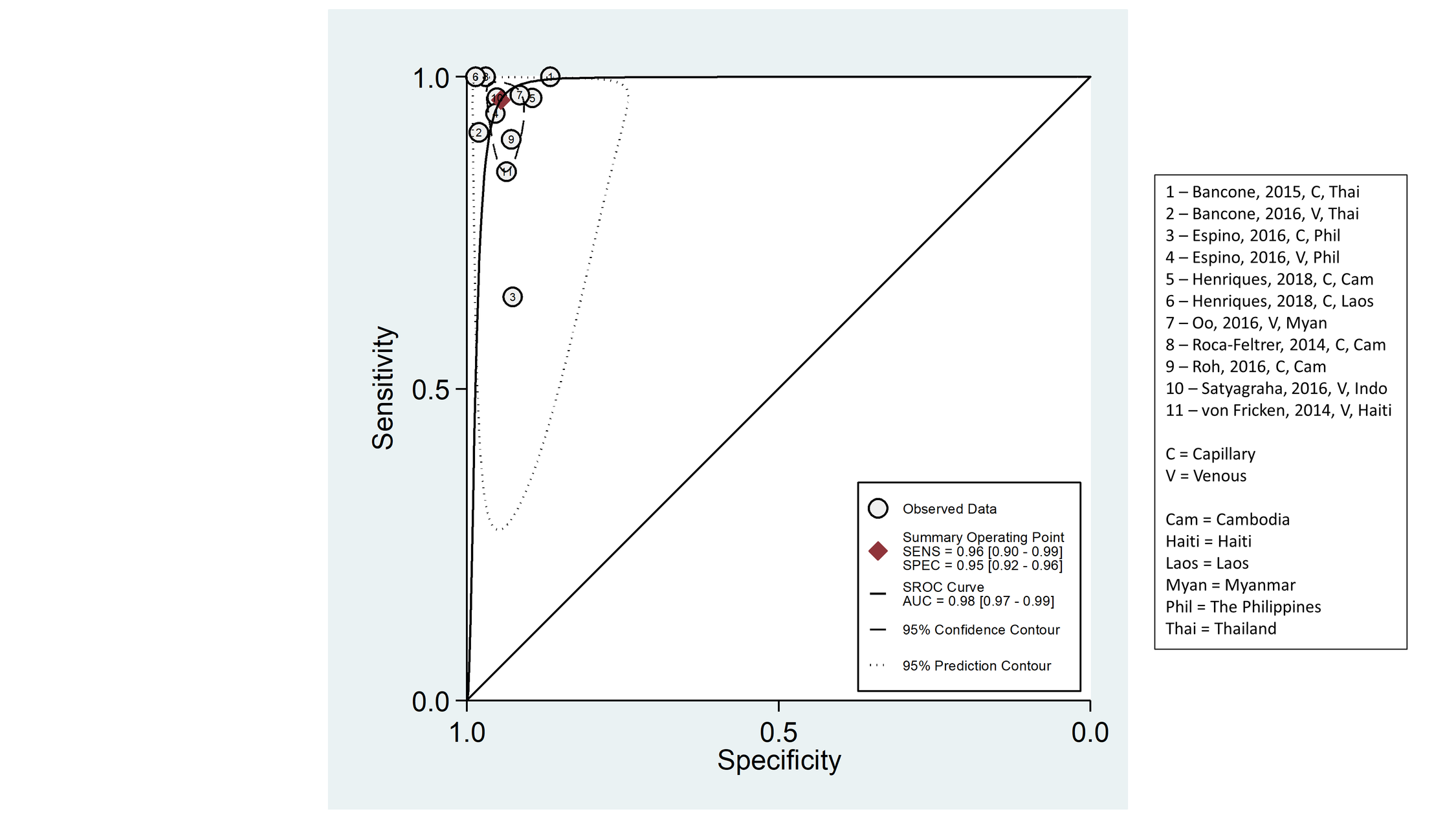

Supplement: S1 Fig — ROC, Receiver Operating Characteristics curve. (TIF) [file pmed.1002992.s005.tif]

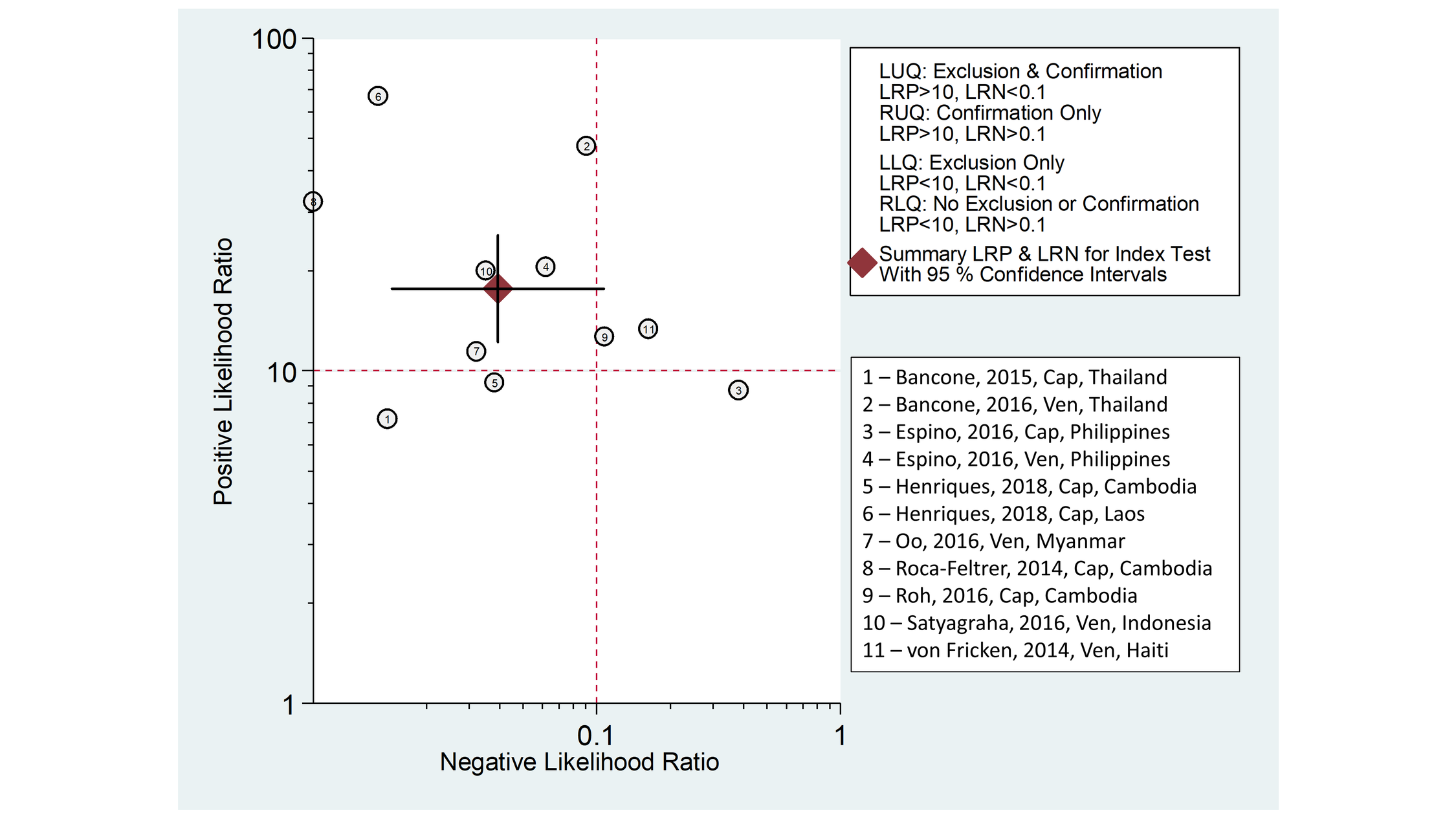

Supplement: S2 Fig — (TIF) [file pmed.1002992.s006.tif]

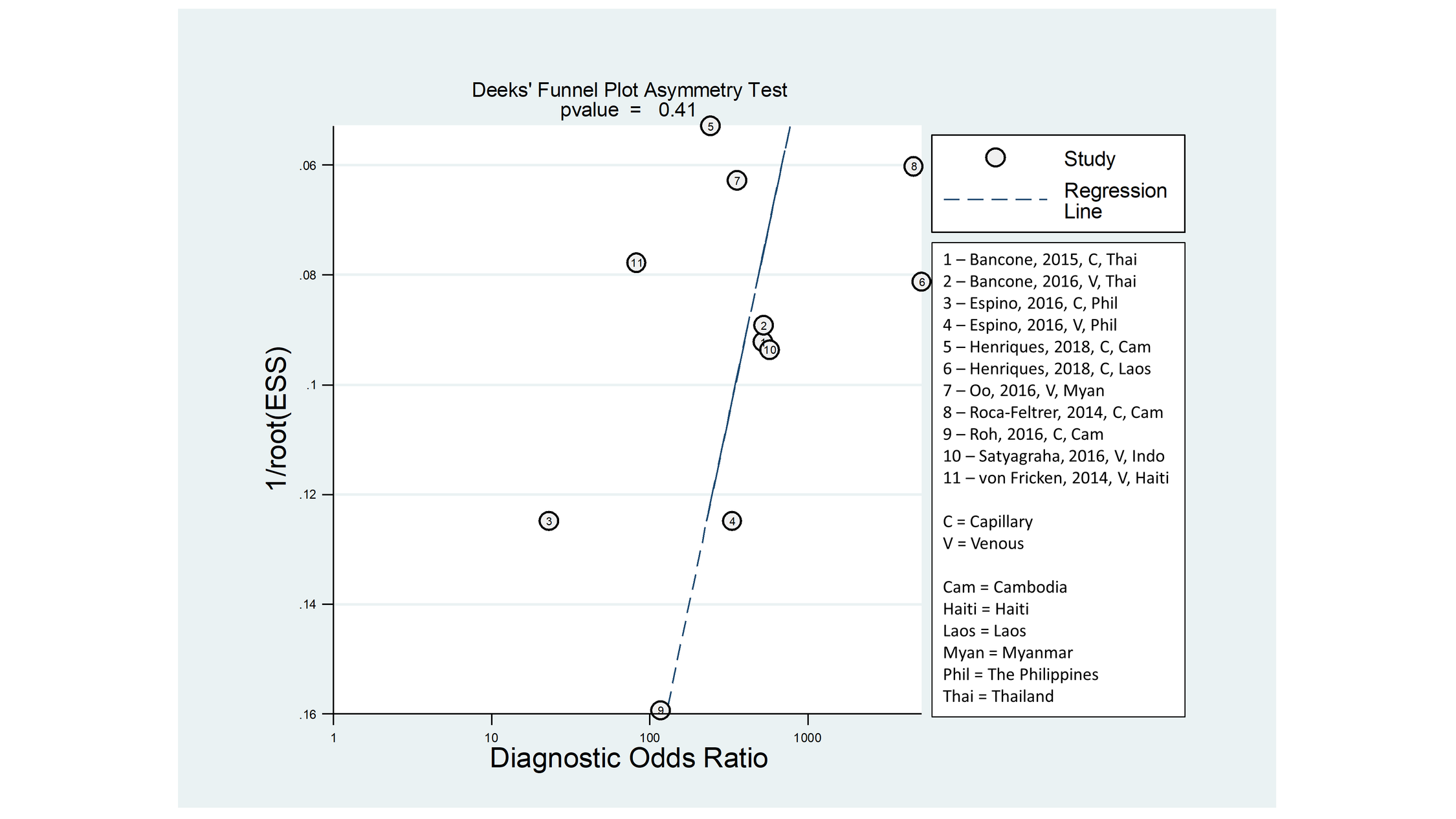

Supplement: S3 Fig — (TIF) [file pmed.1002992.s007.tif]

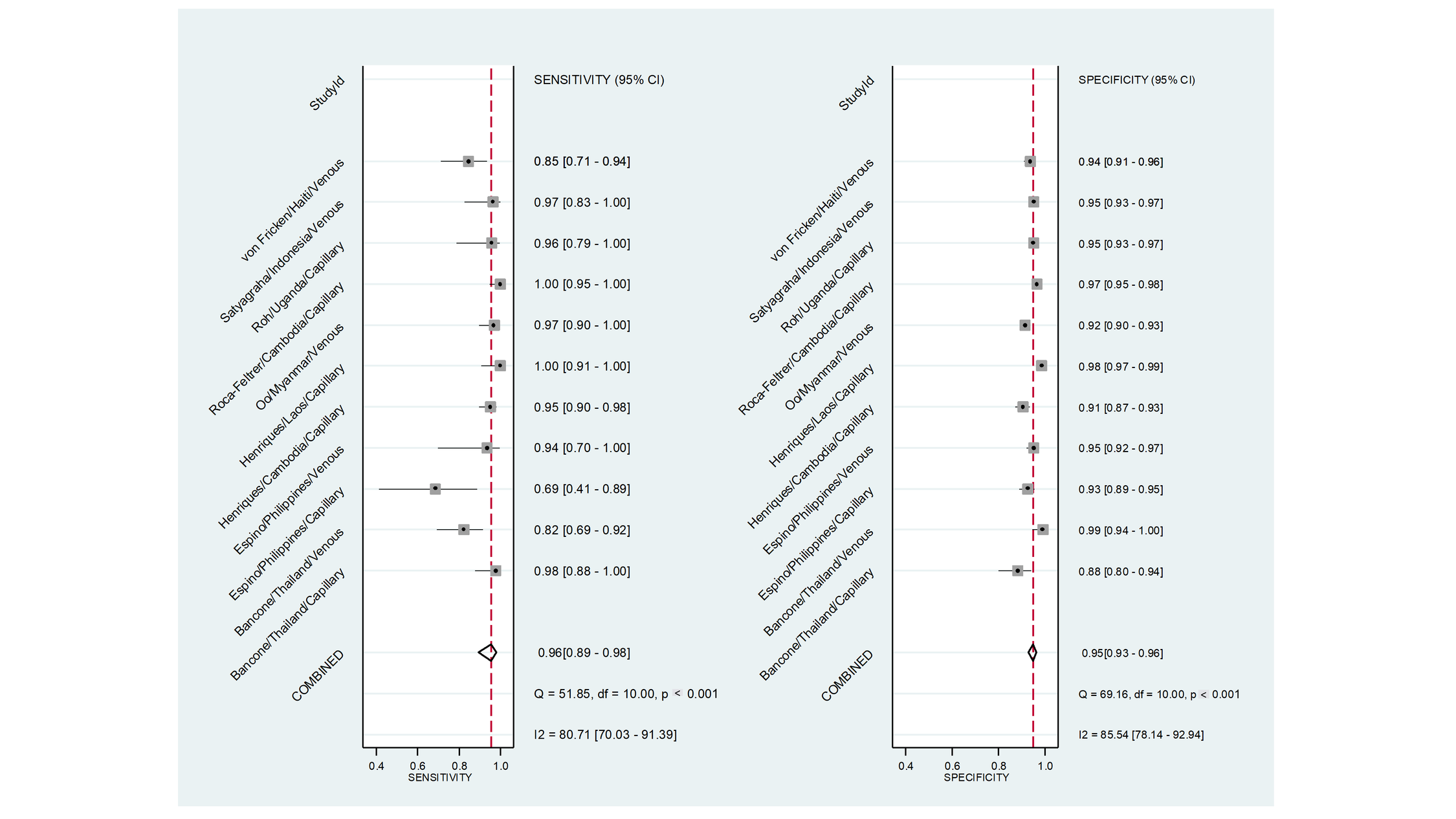

Supplement: S4 Fig — Threshold for G6PDd is calculated based on the pooled AMM. (TIF) [file pmed.1002992.s008.tif]

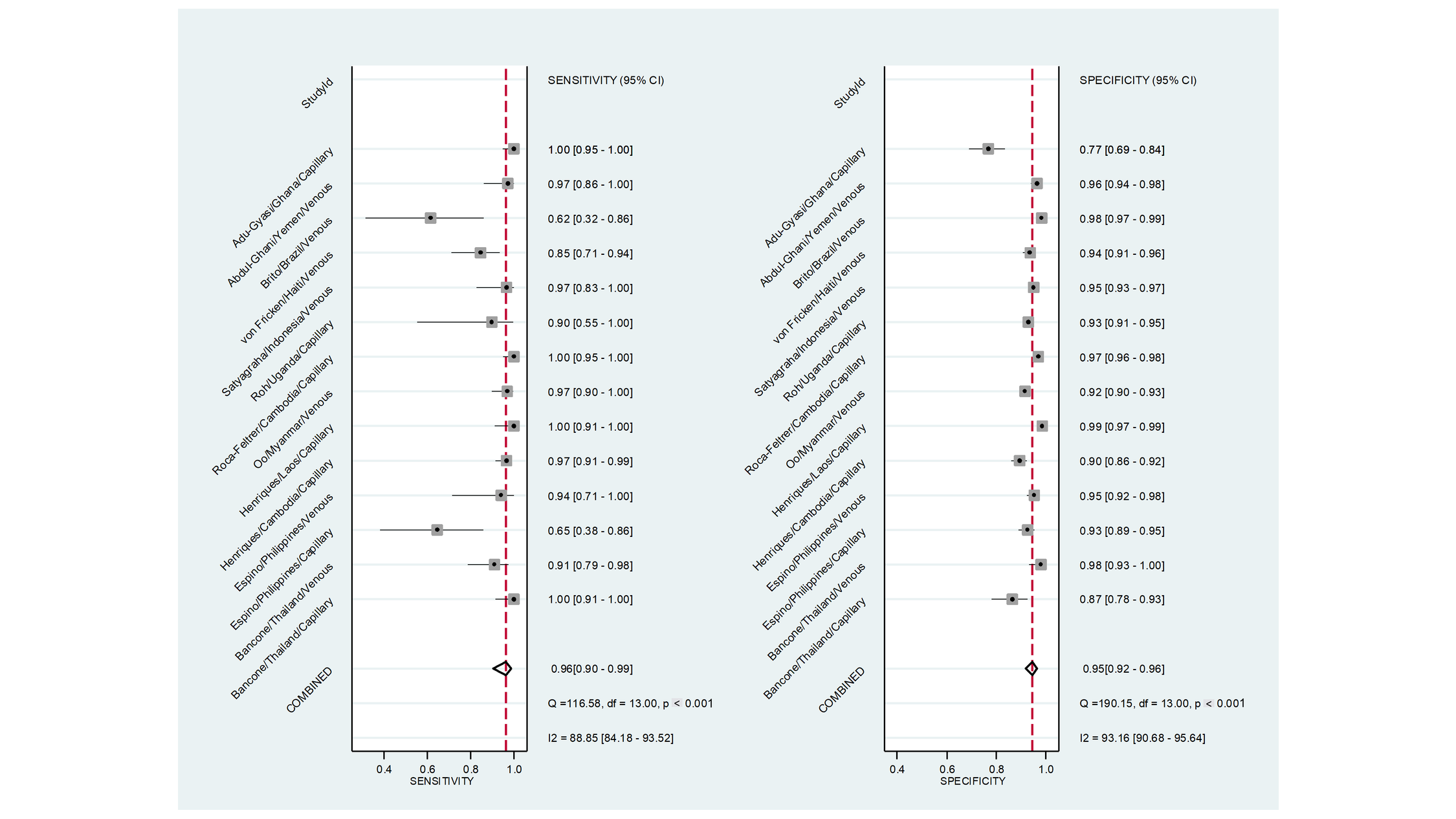

Supplement: S5 Fig — Threshold for G6PDd is based on definitions provided from aggregate data (first 3 studies from top) and calculated based on the site-specific AMM for IPD (all other studies). Study ID is identified by first author, country of sample collection, and type of blood used. (TIF) [file pmed.1002992.s009.tif]
