# Supplementary material for: Performance of the Access Bio/CareStart rapid diagnostic test for the detection of glucose-6-phosphate dehydrogenase deficiency: A systematic review and meta-analysis
Source: PLoS Med. 2019 Dec 13;16(12):e1002992. doi: 10.1371/journal.pmed.1002992 (PMC6910667; doi:10.1371/journal.pmed.1002992)
Supplement: Attachment — Submitted filename: Response to rev. 0.1.docx [file pmed.1002992.s010.docx]

***Line numbers refer to the clean manuscript***

**Reply to reviewer comments**

1. **Editors requests:**

**1.- In addition to the data statement, it would be helpful to provide a list of the data contacts for the included studies in a supplemental file.**

We have added this as table S3.

**2. Please revise your Title to PLOS Medicine style, placing the study design at the end, after a colon “Performance of the Carestart rapid diagnostic test for the detection of glucose 6 phosphate dehydrogenase deficiency: a Systematic review and meta-analysis”**

We have revised the title as requested: *Performance of the Accessbio/Carestart rapid diagnostic test for the detection of glucose 6 phosphate dehydrogenase deficiency: a systematic review and meta-analysis*

**3. PLOS style does not permit trademarks, please remove the (TM) in the title and throughout the manuscript.**

We have removed the Trade Mark text as requested. Please note, that this does not show up in the track change manuscript due to the formatting.

**4. Please combine the Abstract “Methods” and “Results” sections into one section, titled “Methods and Results”.**

We have combined the abstract subheadings as requested

**5. Please include the full dates (day, month, year) of the search in the Abstract, as well as the data sources, eligibility criteria, and synthesis/appraisal methods**

We have added these dates (lines 39 – 42):

*“Articles published on PubMed between 2011 and 27^th^ September 2019 were screened. Articles reporting performance of the standard CSG from venous or capillary samples collected prospectively and considering spectrophotometry as gold standard (using kits from Trinity Biotech PLC, Wicklow, Ireland) were included.”*

**6. Alongside the 95% CIs, please include p-values.**

We have presented performance indicators with 95% confidence interval and now include p-values for all comparisons.

**7. In the last sentence of the Abstract Methods and Findings section, please describe the main limitation(s) of the study's methodology.**

We have added the following sentence to the abstract (Lines 56-58):

*“**Limitations of the study include that the CSG results were generated under research conditions, and hence may not reflect performance in routine settings.”*

**8. At this stage, we ask that you include a short, non-technical Author Summary of your research to make findings accessible to a wide audience that includes both scientists and non-scientists. The Author Summary should immediately follow the Abstract in your revised manuscript. This text is subject to editorial change and should be distinct from the scientific abstract. Please see our author guidelines for more information:**

We have added an Authors summary using the template provided (lines 65 – 82).

**9. Please update your search to the present time.**

We have updated the search until 27^th^ September 2019 (lines 39 and 115). The search returned only one additional paper that did not fit the inclusion criteria. Figure 1 has been updated accordingly.

**10. Please evaluate study quality and risk of bias.**

The risk of bias analysis is now included. We have moved the respective section in the methods section to lines 169 – 170 to streamline the text.

The Results are described in lines 233 – 236:

*“No significant publication bias was detected among included studies (p=0.41), three studies were identified as yielding a high risk of bias, two due to purposive selection of participants (20, 21) and one study due to lack of temperature-controlled spectrophotometry (34) (Fig 4, S3 Fig).”*

**11. Line 97, I believe you mean “sex” rather than “gender”. Please make sure the usage is accurate.**

We have revised the text as requested (lines 43 and 125)

**12.- In the first paragraph of the Discussion and in the Discussion conclusions, please address the study implications without overreaching what can be concluded from the data; the phrase "In this study, we observed ..." may be useful.**

We have revised the respective sentences to be more circumspect.

The first sentence of the discussion (lines 259 – 260) now reads: *“In this meta-analysis we observed an overall sensitivity and specificity of the CSG of more than 95%, the NPV was almost 100% across a wide range of G6PDd prevalences.”*.

The first sentence of the conclusion (lines 324 – 326) now reads: *“The results from this pooled analysis suggest that the CSG provides a reliable method to identify individuals with less than 30% G6PD enzyme activities, based on these findings the test is suitable for introduction into routine treatment prior to PQ but not TQ treatment.”*

**Reviewer #1:**

We thank the reviewer for their positive comments and highlighting the importance of the analysis. We have now included the suggested reference as #24.

Minor Comments:

**1. Lines 133-136: the explanation of likelihood ratios is not very clear and they are described as probabilities in the text. LR+ and LR- are not probabilities - they are ratios, correct interpretation: LR+: quantifies** **how much more likely the positive test of G6PDd is to occur in subjects with the condition compare those without; LR-: quantifies how much less likely the negative test of G6PDd will occur in subjects without disease**

We have changed the respective paragraph accordingly, it now reads: *“**Likelihood ratios are a convenient method to determine the usability of a diagnostic test. In the case of the CSG the positive likelihood ratio (LR+) describes how many times more likely a G6PDd test result occurs in a G6PDd individual compared to the probability that a G6PD normal person is tested G6PDd, the negative likelihood ratio (LR-) is defined as the inverse of this (26).* ” (lines 162 – 166)

**2. Lines 136-137: this statement needs be benchmarked to accepted criteria. (Šimundić et al JIFCC 2009) -. Generally rule is that good test have LR+ > 10 and LR- < 0.1. The results show that the Carestart test satisfies this benchmark.**

We have rephrased the respective sentence in lines 166 -167: *“In general tests with a LR+ above 10 are considered suitable for diagnosis of a condition and a LR- of less than 0.1 is considered suitable to exclude a condition (27).”*

We have also now referenced Simundic et al paper (#27).

**3. Lines 194-196: the authors excluded three studies from the IPD due to not providing study data. Given that three studies did not reply but did meet eligibility criteria, it would be helpful to consider pooling the aggregate study reported outcomes together for 11 studies. This will allow for a comparison with the aggregate samples, given that the three eligible studies contribute about 1280 participants with a higher proportion of malaria patients. That's quite a large number of patients to not include in any of the analyses.**

To address this potential inclusion bias, we have added the requested analysis to the methods section (lines 184 – 189): *“The analysis was again repeated including all data as well as the aggregated data extracted from eligible articles for which individual patients’ data were not available. The definition of TP, TN, FP and FN for articles where no IPD was available was based on definitions applied in the respective studies. Whenever a discrepancy between reported performance and numbers of true and false positives and true and false negatives was found, the latter was considered.”*

The corresponding section under results in the sensitivity analysis (Lines 255-256) now reads: *“When aggregated data were included from the three studies that fulfilled the inclusion criteria, but for which no IPD were available, the performance did not change) (Fig S5). “*

The additional analysis is also reflected in the limitations section, where we have added the sentence: *“Reassuringly, when the analysis was repeated including aggregated data, the test performance did not change.”* (line 319 - 320)

We have also added figure S5 to the supplementary data.

**4. Discussion section: It would be helpful if the authors could benchmark the Carestart diagnostic accuracy results from their IPD against the standard approach of mass spec from the literature. It would also be helpful for the authors to state if there are any of comparable reviews in this area and if so, how do their results compare. If not, they should also state the novelty of their current study.**

Whilst spectrophotometry remains the gold standard, the test is challenging with significant variation between labs and assays. This is addressed in a complementary manuscript currently under review at PLoS Med, but not cited here due to reference rules from PLoS Med (<https://journals.plos.org/plosmedicine/s/submission-guidelines#loc-references>)

Instead we have added the following paragraph to comment on the reference method performance (line 305 – 310):

*“Spectrophotometry remains the gold standard for the diagnosis of G6PDd, and was used as the reference for the current analysis (16). Alternative approaches, such as molecular analysis for G6PD variants correlate poorly with G6PD phenotype, precluding this approach as reliable reference (33, 42, 43). When comparing the Trinity spectrophotometry kits, considered for this analysis, to another spectrophotometry kit (Pointe Scientific, USA), both assays showed a very good correlation (r=0.9799, p<0.001) (44).“*

**5. Limitations section: understand there was a limited data request from the study authors to data providers and hence there were a limited number of covariates. The authors should address this issue as a limitation, and speak to if any potential covariates which have not been captured could influence the results.**

We agree this is an important limitation. To clarify, we have added the following sentence (lines 300 – 304): *“Only a few data variables were collated from all studies and hence our covariate analysis was limited to the hemoglobin concentration, the sex of the participant and country of sample collection. Other factors that may also have influenced the test results could have included batch to batch variability in test kits, the temperature at which the tests were performed, and training and ability of individuals undertaking the tests.“*

**Reviewer #2:**

**1. Lines 63, 64. This seems unhelpfully vague. What are the possible clinical consequences? Also, "can cause"? This seems to infer inconsistency of the effect, i.e., sometimes it does not. If that's true, say so and cite the evidence. Otherwise, the evidence available would lead this reviewer to believe "invariably causes" to be the more appropriate terminology.**

We have changed the sentence (lines 89 - 92) to:

*“Whilst PQ is tolerated in most patients, it can cause haemolysis in patients with glucose-6-phosphate dehydrogenase (G6PD) deficiency, the severity of which is dependent on the underlying genetic variant, the dose of PQ administered and the age of the patients red blood cell (RBC) population (7, 8).”*

**2. Line 65. "More than 215"? How many more? This seems a peculiar expression given the precision of the number.**

We have changed this in line 93:

*“To date 215 genotypes conferring different degrees of…”*

**3. Line 72. They are certainly at significant risk simply by the genetics. The authors perhaps mean "at risk of significant harm".**

This comment refers to lines 99 - 100: Heterozygous females can be at risk of haemolysis but the severity of this can vary considerably, depending on the variant and lyoniztion. We have rephrased to provide clarity: “*Depending on the ratio of G6PD normal to G6PDd RBCs, heterozygous females may be at a risk of severe drug induced haemolysis (13, 14)”* (lines 99 – 100)

**4. Line 77. The expressed affordability of the FST may be challenged. It is about $5 a test just for the reagents and requires a cold-chain and specialised equipment. It is certainly not sufficiently affordable to even referral hospitals in endemic zones, where it is almost never found (because it costs too much).**

We have removed “affordable”. The respective sentence on line 105 - 106 now reads:

*“The fluorescent spot test (FST) is a qualitative alternative, however…”*

**5. Line 226-227. Doubtful that the CSG was engineered to perform poorly above 30% of normal, i.e., "designed". It is likely to be an inherent limitation of the technology.**

We have changed the sentence. Lines 263-264 now reads:

*“The CSG performs best at an approximate 30% cut off activity, however…”*

**Reviewer #3:**

We thank the reviewer for their positive comments.

**1.	The authors used unconditional negative predictive value (NPV), positive (LR+) and negative (LR-) likelihood ratios in the abstract. However, for the general audience, they are not immediately understandable (though they are explained in the main text). Suggest to include a short description of what the meanings of the results are.**

We explain our findings in the discussion section of the abstract, where it reads: *“Its high NPV suggests that the test is suitable to guide PQ treatment, the high LR+ and low LR- render the test suitable to confirm and exclude G6PDd” (lines 59 – 61)*

**2.	Line 76: the gold standard quantitative method is expensive - it should be clarified that there are many G6PD quantitative kits available, but the one from Trinity is much more expensive.**

The availability of the quantitative tests varies considerably and novel hand-held devices are now reaching the market. From a public health perspective, spectrophotometry is too expensive for routine care in most malaria endemic areas. This is due to direct costs as well as lab infrastructure and training costs.

We believe that the statement on lines 102-105 reflects this: *“The gold standard method for measuring G6PD activity is quantitative spectrophotometry (15, 16), but this method is expensive and requires laboratory facilities that are often unavailable in malaria endemic communities, especially in remote areas.”*

**3.	Some studies compared the assay results from both venous and capillary blood. I wonder whether there is any basis suggesting that the two sources would be different in G6PD activity?**

We agree that this is an important caveat. In the discussion, we highlight this issue on lines 274 to 281: *“Bancone et al. previously compared CSG results from paired venous and capillary samples, with 11% discrepancy between samples, with a sensitivity at the 30% threshold of 100% in capillary samples compared to 89% in venous samples (21). In the same study the authors also correlated their findings with hematological parameters and found that red blood cell concentration, hemoglobin, hematocrit, mean corpuscular volume, and platelet count varied slightly between venous and capillary samples, however they concluded that these differences were unlikely to have a major effect on the performance of the CSG (21).”*

**4.	In sensitivity analysis, the sensitivity for males was significantly higher than for females. Is there a potential explanation for this?**

This is an important finding that we offer an explanation for in the discussion (lines 263-271): *“The CSG performed significantly better in males compared to females. The CSG performs best at an approximate 30% cut off activity (20), however the absolute cut-off of the CSG and the absolute cut-off calculated from spectrophotometry do not necessarily match. Since males are either hemizygous normal or deficient their enzyme activity will be either below or well above the 30% cut off, and thus small discrepancies between the two thresholds will not affect the calculated performance. However, females can be either homozygous or heterozygous for the G6PD gene, the latter manifesting phenotypically with enzyme activities ranging from almost normal to G6PD deficient (12-14). Hence in heterozygous females small differences between the inherent test and the calculated cut-off activity will affect the tests performance adversely.”*

*Minor Comments:*

**1.	For consistency with published literature, please use PQ as abbreviation for primaquine and use it throughout the text (see lines 74, 254).**

We have revised the text accordingly changing Pq to PQ and Tq to TQ throughout.

**2.	I suggest to use CareStart in the abstract, since it is difficult to see why Carestart is abbreviated into CSG.**

The abbreviation CSG (Carestart screening test for G6PD deficiency) had been used in at least two earlier publications and we would like to stick to this abbreviation. But we have now clarified the term in the abstract in line 36 and in the introduction (line 108).

**3.	In the abstract Introduction section, "a qualitative lateral flow assay from Carestart, USA" should be "CarestartTM G6PD RDT, a qualitative lateral flow assay from AccessBio, USA".**

We note the journals reluctance to use Trade Marks. We now stated the following in lines 35-38:

*“This systematic review and individual patient meta-analysis assessed the utility of a qualitative lateral flow assay from Accessbio/Carestart, USA, (CSG) for the diagnosis of G6PDd compared to the gold standard spectrophotometry (PROSPERO: CRD42019110994).”*

**4.	Line 222, use abbreviation NPV or spell out it in the entire text.**

We have revised the text accordingly on line 260.
